# Hygienic Rules

**Published:** 1873-01

**Authors:** 


HYGIENIC RULES.—Never eat when much fatigued; wait until rested.

Never eat just before you expect to engage in any severe mental or physical exercise.

Never eat while in a passion, or when under any great mental excitement, depressing or elevating.

Never eat just before taking a bath of any kind, or just before retiring at night.

Never eat between regular meals.

